# Obstructive Jaundice

**Published:** 1902-01-25

**Authors:** 


### HOSPITAL CLINICS AND MEDICAL PROGRESS.

### OBSTRUCTIVE JAUNDICE.

AT the last meeting of the Medical Society, Mr. Mayo Robson gave the results of his experience in regard to the operative treatment of obstructive jaundice based upon upwards of 200 cases. He chiefly concerned himself with an endeavour to throw some light upon the problem in what cases to operate, a matter of great clinical importance in view of the fact that, while in some cases a cure may be obtained even in apparently desperate conditions, in other cases, if only they can be diagnosed with certainty, no operation can do the slightest good. He thinks that it should be distinctly recognised that no patient ought to be allowed to die of obstructive jaundice without the question of surgical interference being considered, and further that this question ought to be raised at a time when operation may be undertaken, if called for, with a reasonable hope of success. In the following cases, he says, surgery holds out a good prospect of cure :—(1) Common duct cholelithiasis ; (2) chronic pancreatitis ; (3) simple stricture of the common bile duct ; (4) inflammatory adhesions causing pressure on, or stenosis of the hepatic or of the common bile ducts ; (5) hydatid disease of the liver, pressing on or discharging into the bile ducts. Thus one may summarise the question of treatment by saying (*a*) that if the diagnosis be doubtful an exploratory operation is advisable, providing the general condition of the patient renders it probable that such a procedure *per se*, apart from what is to follow, will not hasten death ; (*b*) that if malignant disease be positively diagnosed operation can, with some few exceptions, where it is possible to completely remove it, do but little or no good, or at best can probably only prolong life for a short time and (*c*) that if gall-stones, or in fact any of the five conditions enumerated above can be diagnosed, operation is decidedly advisable if the patient be at all in a condition to bear it. It is quite obvious from the remarks made by Mr. Mayo Robson that the whole difficulty, putting aside all considerations of technique and operative skill, ranges around the question of diagnosis, and that many brilliant results have been obtained by operating in "doubtful cases," mostly in cases which have been presumed to be cancerous, but have turned out to be either gall-stones or cases of chronic pancreatitis. It will be apparent from this how very important is this question of diagnosis, and although this is not the place for a treatise on the subject, we may usefully extract from Mr. Robson's remarks various maxims which he has formulated as the outcome of his very considerable experience.

A painless onset of chronic jaundice must always give rise to the suspicion either of chronic catarrh, dependent upon cancer of the liver or on occlusion of the hepatic or common bile duct by growth, and if this be associated with distension of the gall bladder and rapid loss of weight and strength, a cancer of the head of the pancreas will probably be found. On the other hand, a history of an attack of pain in the upper abdomen, followed soon by jaundice and preceded by so-called spasms, either recently or at some remote period, is strongly suggestive of cholelithiasis. In the latter case the jaundice will probably be less intense than in the former, and an intermittent fever with chills and sweats will probably either be present or have been noticed at some stage of the disease.

Enlargement of the liver is much more common in obstruction due to cancer than in that from gall-stones, though it may be present in either.

The presence of ascites is suggestive of malignant disease, although to this there are exceptions. As a rule the presence of ascites negatives any radical operation on the bile ducts.

Time and history will clear up certain points ; for instance, the jaundice of gall-stones is rarely continuously the same, but increases or diminishes from time to time, whereas the jaundice of obstruction due to growth steadily increases or tends to become absolute, especially in cancer of the bile duct or the head of the pancreas. Again jaundice, with malignant disease, runs a very short course ; whereas, that from gall-stones or other simple cause may go on for several years. But it must be remembered that there may be both cancer and gall-stones, and that the combined disease may form a tumour before the onset of the jaundice.

Ague-like symptons more frequently accompany gall-stones in the common duct than malignant disease of the ducts or of the head of the pancreas, although to this rule there are exceptions.

A rigid right rectus abdominis, and tenderness one inch above and to the right of the umbilicus are as suggestive of gall-stones as is McBurney's tender point of appendicitis. Even after the abdomen is opened doubt may arise as to the interpretation of the appearances presented, and the surgeon is urged, on the one hand, before seriously interfering with adhesions and thus becoming committed to the following out of a dangerous and futile operation, to raise the abdominal wall and carefully examine the liver and adjoining parts so as to be assured of the absence of secondary nodules of cancer ; and, on the other hand, not to be too ready to condemn a patient in whom the head of the pancreas may be found swollen and harder than normal, for the swelling may be a chronic pancreatitis and may be curable by operation, as has now been proved in many instances. The feeling is strongly impressed upon us by Mr. Mayo Robson's address that, although there are some conditions which, when they can be certainly diagnosed, should exclude operation, whenever there exists any doubt at all operation should be considered and perhaps performed. Some of his most successful cases appear to have been the most full of doubt until operation cleared up the meaning and the origin of the symptoms.

